# Adenosine-related small molecules show utility of recall antigen assay to screen compounds for off-target effects on memory T cells

**DOI:** 10.1038/s41598-021-88965-3

**Published:** 2021-05-05

**Authors:** Eden Kleiman, Gloria Sierra, Binchen Mao, Dennie Magcase, Marybeth V. George, Pirouz M. Daftarian

**Affiliations:** 1 JSR Life Sciences, 1280 North Mathilda Avenue, Sunnyvale, CA 94089 USA; 2 Crown Bioscience Inc, Room 303, Building A6, No. 218, Xinghu Street, Industrial Park, Suzhou, 215000 Jiangsu Province People’s Republic of China; 3 Present Address: Crown Bioscience Inc., 16550 W Bernardo Dr Building 5, Suite 525, San Diego, CA 92127 USA; 4 Present Address: Department of Thoracic Head and Neck Medical Oncology, MD Anderson Cancer Center, The University of Texas, 1515 Holcombe Blvd, Houston, TX 77005 USA

**Keywords:** Biological techniques, Cancer, Cell biology, Drug discovery, Immunology

## Abstract

Extracellular adenosine suppresses T cell immunity in the tumor microenvironment and in vitro treatment of memory T cells with adenosine can suppress antigen-mediated memory T cell expansion. We describe utilizing the recall antigen assay platform to screen small molecule drug off-target effects on memory T cell expansion/function using a dosing regimen based on adenosine treatment. As a proof of principle, we show low dose GS-5734, a monophosphoramidate prodrug of an adenosine analog, does not alter memory T cell recall at lower doses whereas toxicity observed at high dose favors antigen-specific memory T cell survival/proliferation over non-specific CD8^+^ T cells. Conversely, parent nucleoside GS-441524 at high dosage does not result in cellular toxicity and reduces antigen-specific T cell recall in most donors. Despite similar chemical structure, these drugs displayed opposing effects on memory T cell expansion and viability highlighting the sensitivity of this assay setup in screening compounds for off-target effects.

## Introduction

The recent emergence of SARS-CoV-2 has redirected research efforts towards understanding the adaptive immune response and enhancing vaccine development targeting this virus. Recent evidence reinforces the crucial role of a strong T cell immune response in SARS-CoV-2 disease^1, 2^ control while also illustrating the link between disease progression and the inability to sequester appropriate T cell help^3–6^. GS-5734 (Remdesivir) is a monophosphoramidate prodrug of an adenosine analog currently being utilized for therapeutic use in SARS-CoV-2 infections^7–9^ due to its ability to potently inhibit SARS-CoV-2 viral load by blocking viral activity of RNA-dependent RNA polymerase (EC_50_ range of 0.77 µM to 23.15 µM in SARS-CoV-2 infected Vero E6 cells)^10–12^. Remdesivir has previously shown effectiveness against MERS, SARS, Ebola and other viruses^13^ and can prevent SARS-CoV-2-mediated progression to pneumonia in rhesus macaques^14^. It is unclear if the antiviral effects of adenosine-related drugs are related to any potential modulation of T cell immunity as a mechanism of action or off-target effect.

Interestingly, adenosine itself is a suppressive agent in the tumor microenvironment (TME) that depresses cytotoxic T cell activity. Cancer-related hypoxia can elevate extracellular adenosine due to enzymatic ATP catabolism, particularly by the rate limiting ectonucleotidase enzyme CD73. Local fluctuations in adenosine can cause TME concentrations to reach into the hundreds of micromolars^15,16^. Given the importance of T cell mediated immunity in counteracting viral infections such as SARS-CoV-2, assays to screen small molecule drugs (e.g. anti-virals) for potential off-target effects on memory T cells would be helpful. We show that sequential high dose adenosine addition can potently suppress antigen-specific memory T cell expansion and that this dosing regimen can be used to test other drugs for off-target effects on antigen-specific T cell recall and functionality.

GS-5734 at low doses does not inhibit memory T cell recall but toxicity is observed at high doses, consistent with the reported CC_50_^10^, coinciding with favored expansion/survival of antigen-specific T cells over the general CD8^+^ T cell pool_._ Surprisingly, high dosage of parent nucleoside GS-441524 displayed mostly inhibitory effects on antigen-specific T cell recall with no adverse effect on general cell viability. Using surface CD137 expression as an activation read-out, high dose GS-5734 and GS-441524 displayed varying effects that were both donor- and peptide-dependent. This assay is a novel application of the antigen recall assay to screen small molecule drugs for off-target effects on both CD8^+^ as well as CD4^+^ memory T cell response.

## Materials and methods

### Recall antigen assay

The recall antigen assay spanned a duration of 7 days. On day 0, donor PBMCs (purchased from Astarte Biologics, now part of Cellero, Lowell, MA) were washed twice after thaw and resuspended at 5 × 10^6^ viable cells per mL in AIM V medium (ThermoFisher Scientific, Waltham, MA, Cat # 12055091) with 5% human AB serum (Millipore Sigma, Burlington, MA, Cat # H3667). Control unstimulated cells were first plated in a round-bottom 96-well plate, 100 µL per well (500,000 cells). For the remainder of cells, 10 µg/mL peptide (final concentration unless otherwise stated) was added to cells to create a cell/peptide mixture. 100 µL (500,000 cells) of cell/peptide mixture was added to appropriate wells. NLVPMVATV peptide was purchased from Astarte Biologics. IVTDFSVIK, AVFDRKSDAK, and ATVQGQNLK peptides were purchased from MBLI (Chicago, IL). On day 1 of the recall antigen assay, each well was supplemented with 100 µL complete medium, 50U/mL IL-2 final concentration (Millipore Sigma, Cat # 11147528001) with or without treatments (i.e. adenosine, GS-5734 and GS-441524) for a final well volume of 200 µL. On days 3 and 5, 100 µL volume from each well was removed without disturbing cell pellet. This was replaced with fresh complete IL-2 supplemented media (50 U/mL final concentration), with or without treatments so that total well volume was maintained at 200 µL. Readout for recall antigen assay was flow cytometry on day 7. Tetramers were used in flow cytometric analysis that corresponded to the peptide used for stimulation. Tetramer positive cells correspond to memory T cells that expanded in response to peptide stimulation on day 0. For adenosine-treated wells, 1 mM adenosine (Millipore Sigma, Cat # A9251) was given on day 1 and 0.5–0.75 mM adenosine was given on days 3 and 5. Adenosine was dissolved in complete media unless otherwise noted. GS-5734 and GS-445124 (Cayman Chemical, Ann Arbor, MI) were dissolved in Hybri-Max DMSO (Millipore Sigma, Cat # D2650). Adenosine was also prepared in Hybri-Max DMSO and used as a control in initial experiments although at much lower concentration than adenosine dissolved in complete medium. Adenosine deaminase (ADA) (Cat #10102105001) and Inosine (Cat #I4125) were both purchased from Millipore Sigma. Inosine was prepared in complete media. Tofacitinib (Selleckchem, Cat # CP-690550) was prepared in DMSO.

### 48-h memory T cell re-activation

For 48-h stimulation of previously expanded PBMCs, the ratio of expanded to unexpanded (same donor PBMCs) was 2:1 with 500,000 cells total per well. 1 mM adenosine was added to appropriate wells at the time of peptide stimulation. The readout for 2-day peptide-pulsed samples was flow cytometry using both surface staining for tetramer and intracellular cytokine staining.

### Bead-based proliferation assay

Donor PBMCs were incubated with 5 µM CellTrace Blue Cell Proliferation kit (ThermoFisher, Cat # C34574) proliferation dye and then stimulated with anti-CD3/anti-CD28 beads (ThermoFisher, Cat # 11132D) in a 96-well round-bottom plate. Adenosine was added to appropriate wells day 0 at 1 mM final concentration and again on day 1 at 0.5 mM final concentration. Proliferation was assessed 4 days post-stimulation for proliferation dye dilution using flow cytometry.

### Surface and intracellular staining

PBMCs were first incubated with 50 nM Dasatinib (Selleck Chemical LLC, Cat # S1021) in complete medium for 15 min at 37 °C. Dasatinib is used to prevent TCR downregulation and enhance tetramer staining^17^. Cells were washed with FACS buffer (0.5% BSA in PBS) and then stained with antibody/tetramer cocktail (in FACS buffer) for 30 min at room temperature. Cells were then washed and resuspended in FACS buffer with viability dye 7-AAD (MBLI, Code # FP00020050) except for the experiment using donor #4 and #5 PBMCs which used Fixable Viability Dye eFLuor 780 (ThermoFisher, Cat #65–0865-14). For Annexin V (BioVision Inc., Milpitas, CA, Cat # K101-100) and 7-AAD co-staining, both were added at the same time in 1X Annexin V binding buffer.

For intracellular stains, cells were washed with PBS twice following surface stain. Cells were then fixed, permeabilized and stained in permeabilization buffer according to manufacturer’s guidelines. Intracellular staining was performed using either the True-Nuclear Transcription Factor Buffer Set (Biolegend, San Diego, CA, Cat # 424401) or CytoFix Fixation buffer/FACS Permeabilizing solution 2 (BD Biosciences, San Jose, CA, Cat # 554655/340973). For True-Nuclear Transcription Factor Buffer Set the fix and permeabilization reagents were True-Nuclear 1X Fix Concentrate and True-Nuclear 1X Perm Buffer, respectively. Alternatively, cells were fixed using CytoFix Fixation buffer and permeabilized using permeabilizing solution 2. After intracellular staining, cells were washed twice in PBS and resuspended in PBS. For intracellular cytokine staining, 5 μg/mL brefeldin A (Millipore Sigma, Cat # B7651) was added 4 h prior to surface staining (3 h 45 min prior to Dasatinib).

Detection reagents used are as follows: CD3 FITC (Code # FP10255010), CD4 APC (Code # FP10328010), HLA-A*02:01 CMV pp65 tetramer NLVPMVATV-PE (Code # TB-0010–1), T-Select HLA-A*11:04 EBV EBNA3B 416–424 tetramer IVTDFSVIK-PE (Code # TS-M029-1), HLA-A*11:01 EBV EBNA3B tetramer AVFDRKSDAK-PE (Code # TS-M028-1), HLA-A*11:01 CMV pp65 tetramer ATVQGQNLK-PE (Code # TB-M012-1) were from MBLI. CD8 BV510 (Cat # 344,732), TIM-3 BV711 (Cat # 345,024), CD137 BV421 (Cat # 309,820), CD45RO BV785 (Cat # 304,234), CD14 BV785 (Cat # 301,840), CD11b BV711 (Cat # 301,344), and CD28 BV711 (Cat # 302,948) were from Biolegend. PD-1 BV421 (Cat # 564,323) and IFNγ BV421 (Cat # 562,988) were from BD Biosciences. LAG-3 APC (Cat # 17–2239-42), PD-L1 PE (Cat # 12–5983-42) and PD-L1 PE (Cat # 12-5888-42) were from (ThermoFisher—eBioscience). TOX APC (Cat # 130–118-474) was from Miltenyi Biotec (Bergisch Gladbach, Germany).

### Analysis software

Statistical analysis was performed using GraphPad Prism software (GraphPad, San Diego, CA). All bar graph data presented is shown as mean with standard deviation. Flow cytometry analysis was performed using FlowJo software (BD Biosciences).

## Results

### Sequential high-dose adenosine Inhibits memory T cell recall

**Figure 1:**
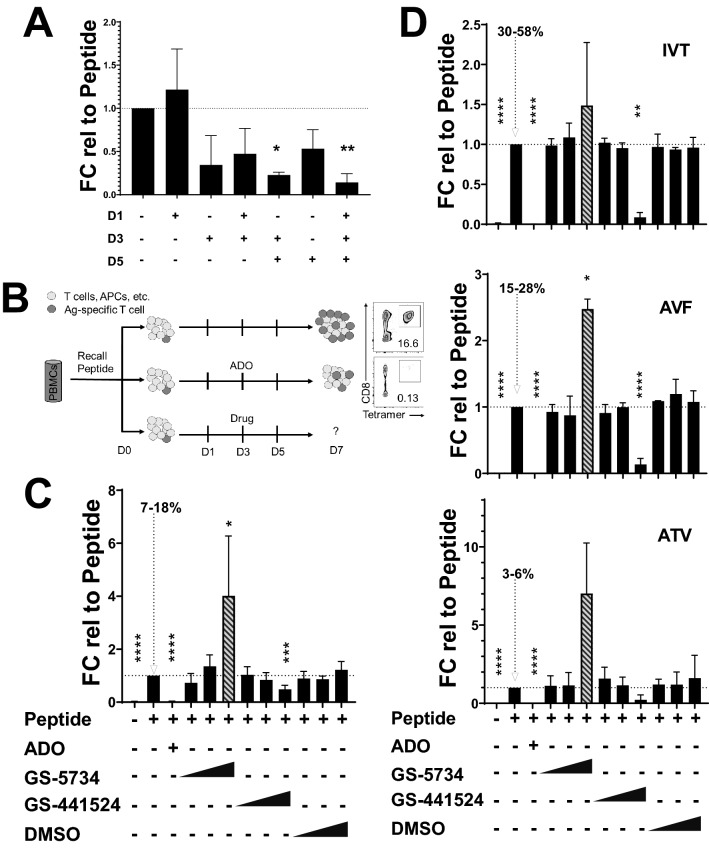
Adenosine-based recall antigen assay. (**A**) HLA-A*02:01 donor #1 PBMCs treated either with peptide alone (CMV pp65 NLVPMVATV) or peptide plus adenosine (ADO) on indicated days (x axis) and assayed on day 7. ADO treatments on day 1 were 1 mM final concentration while day 3 and day 5 ADO treatments are at a final ADO concentration of 0.5–0.75 mM. Data are representative of 3 independent experiments, except for the D3 (n of 2) and displayed as percent tetramer fold-change (FC) relative to peptide-only treated cells within each respective experiment. Dotted line used for comparison. (**B**) Assay setup whereby donor PBMCs are treated with ADO days 1, 3 and 5 and assayed for CD8^+^ tetramer positive T cells. ADO results in significantly reduced tetramer positive T cell expansion. Drug arm uses the same dosing regimen as ADO. (**C**) Donor #1 PBMCs were treated with NLVPMVATV or (**D**) HLA-A*11:01 donor #3 PBMCs treated with EBV EBNA-3B peptide IVTDFSVIK, EBV EBNA4 peptide AVFDRKSDAK, or CMV pp65 peptide ATVQGQNLK. The percent positive range of tetramer positive cells (among live T cells) for peptide-only stimulated wells is indicated with dotted arrow. GS-5734 and GS-441524 (parent nucleoside of GS-5734) were used at increasing doses of 500 nM, 5 µM and 50 µM. 50 µM GS-5734 treatment is indicated with striped bar to denote its high toxicity. DMSO was administered at the same concentrations as drugs; 0.001%, 0.01% or 0.1% final. ADO resulted in almost complete suppression of peptide-stimulated antigen-specific T cell expansion (third lane of each figure). Data are representative of 4 (**C**) or 3 (**D**) independent experiments, each experiment derived from a separate cryovial (same donor and lot number) and each condition was performed in duplicate or triplicate. (**A**,**C**,**D**) Ratios of all combinations of treated sample relative to peptide-only treated sample were calculated and median ratio values for each experiment are plotted. Median values were log transformed for one-way ANOVA and subsequent Dunnett’s multiple comparison test using peptide-only treated samples as the control group. *, adj p value ≤ 0.05; **, adj p value ≤ 0.01; ***, adj p value ≤ 0.001; ****, adj p value ≤ 0.0001.

We employed a reductionist approach to simulate one aspect of the TME environment by incorporating a series of high dose adenosine treatments during the course of the recall antigen assay to specifically suppress antigen-specific memory T cell expansion without loss of PBMC viability. We adopted a flow cytometry-based tetramer readout to measure of CD8^+^ antigen-specific T cell expansion on day 7 post-peptide stimulation. Adding adenosine on day 1, 3 and 5 gave the most consistent suppression of expansion, although adenosine treatment on days 3 and 5 also inhibited expansion suggesting an optimal window for adenosine mediated suppression (Fig. 1A). All subsequent experiments were performed administering adenosine (or drug) on days 1, 3 and 5 (Fig. 1B). This assay format has utility in screening drugs targeting the adenosinergic signaling pathway, particularly A2AR receptor antagonists (unpublished internal data) but is applicable to any other pathway. Adenosine and adenosine analogs have typically been administered as in vitro screens to assess the ability of compounds to reverse cytokine suppression or short term cAMP induction^18,19^. To our knowledge this is the first reported use of adenosine and other small molecules to screen human memory T cells during peptide-induced expansion for off-target effects (Fig. 1B).

Initially, we sought to characterize the mechanism of adenosine-mediated suppression on memory T cell proliferation. Adenosine inhibited T cell proliferation of anti-CD3/anti-CD28 stimulated PBMCs (Fig. S1) consistent with prior results^20^. Adenosine, at the same high dose, also inhibited peptide-induced antigen-specific T cell expansion (Fig. 1C,D discussed below). Antigen-specific T cells that emerged from adenosine treatment stained positive for Ki67 to the same degree as non-adenosine treated samples (Fig. S2). Furthermore, cell viability was not adversely affected by high dose adenosine (Fig. S3-5). This suggests that most, but not all, memory T cell expansion was inhibited by persistent high dose adenosine.

Adenosine metabolites are unlikely to be mediating the observed suppression as enzyme supplementation with adenosine deaminase (ADA) restored memory T cell recall (Fig. S6A). Treatment with adenosine metabolite inosine (at equivalent concentrations to adenosine) resulted in no diminution of memory T cell expansion and in fact was slightly higher consistent with recent evidence that CD8^+^ T cells can use inosine as an alternative carbon source to fuel expansion^21^ (Fig. S6B). Adenosine also displayed limited impact on memory T cell co-inhibitory receptor surface expression as PD-1 and LAG-3 were not significantly altered. However, adenosine caused downregulation of TIM-3. Bystander CD8^+^ T cells (tetramer negative) were more susceptible to adenosine-mediated co-inhibitory receptor downregulation (Fig. S7). Adenosine treatment also affected co-inhibitory receptor expression on non-lymphoid cells. Short-term adenosine (no peptide) treatment significantly increased the percentage and surface expression of PD-1 ligand PD-L1/PD-L2 in CD14^+^ CD11b^-^ cells (Fig. S8). However, adenosine did not negatively affect short term IFNγ cytokine production or induction of polyfunctional human effector memory CD8^+^ T cell marker TOX (Fig. S9)^22^ on expanded memory T cells. Together, these data suggest that in our system, high dose adenosine primarily suppresses memory T cell proliferation with limited effect on activation.

### Chemically similar anti-viral drugs have divergent effects on memory T cell proliferation at high dose

Recent evidence has suggested GS-5734 as a promising drug in the fight against SARS-CoV-2. We chose to test this prodrug and GS-441524 since they were similar in structure to adenosine. Characterized PBMCs containing memory T cells responsive to CMV pp65 peptides or EBV peptides were utilized in these studies as surrogates for how other antigen-specific memory T cells would behave. Data from HLA-A*02:01 donor #1 PBMCs stimulated with CMV pp65 NLVPMVATV peptide shows that 500 nM and 5 µM GS-5734 did not alter memory T cell expansion (Fig. 1C and S10). High dose GS-5734 treatment at 50 µM was associated with toxicity, consistent with its reported CC_50_^10^, particularly towards CD8^+^ T cells (Fig. S3, 5). However, 50 µM GS-5734 resulted in a statistically higher percentage of antigen-specific memory T cells relative to non-antigen-specific CD8^+^ T cells (Fig. 1C). Whether this is due to an increased survival advantage of antigen-specific memory T cells or increased proliferation in the absence of competing CD8^+^ T cells is unclear. GS-441524 did not alter antigen-mediated memory T cell proliferation at 500 nM and 5uM but significantly reduced expansion at 50 µM (Fig. 1C) with minimal associated cellular toxicity (Figs. S3, 5). Nonspecific adenosine receptor agonist NECA and A2AR-specific agonist CGS-21680 have been reported to modestly reduce murine T cell proliferation when stimulated with anti-CD3 monoclonal antibody^23^. However, donor #1 PBMCs treated with NECA and the CGS-21680 displayed negligible effects on proliferation (Fig. S10) at the doses used in our system.

We obtained similar results with GS-5734 and GS-441524 using a second donor harboring a different HLA allele. HLA-A*11:01 donor #3 PBMCs were treated separately with either EBV EBNA-3B peptide IVTDFSVIK, EBV EBNA4 peptide AVFDRKSDAK or CMV pp65 peptide ATVQCQNLK. High dose GS-5734 displayed toxicity, and consistently resulted in an upwards trend in the percentage of antigen-specific memory T cells among the T cell pool reaching statistical significance in the AVF-peptide treated samples (Fig. 1D). Also consistent with data from donor #1, high dose GS-441524 lowered antigen-specific memory T cell recall (Fig. 1D—significantly in two of three cases) without affecting cell viability (Fig. S4).

These data were further confirmed using two additional donor PBMCs (HLA-A*02:01 donor #4 and #5). For donors #4 and #5, 50 µM GS-5734 treatment increased both the percentage of antigen-specific memory T cells and overall cell death (Fig. S11A-B). High dose GS-441524 decreased antigen-specific memory T cell recall in donor #4 but increased antigen-specific memory T cell recall in donor #5 suggesting GS-441524 displays donor-dependent effects (Fig. S11 A-B). Expanded memory T cells treated with high dose adenosine, GS-5734 or GS-441524 show high levels of CD28 surface expression (Fig. S11 B,C, E, F) suggesting that the tetramer positive T cells being examined are unlikely to be CD28^low/-^ CD8 Treg cells^24^, but are more likely antigen-experienced memory T cells in the CMV latency phase^25, 26^.

### Assay platform can be used to assess memory T cell activation effects

**Figure 2:**
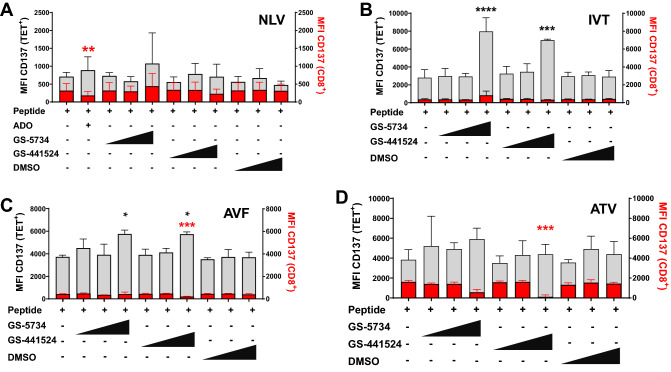
Effect of drug on T cell activation. (**A**) HLA-A*02:01 donor #1 PBMCs and (**B**–**D**) HLA-A*11:01 donor #3 PBMCs were treated and assayed on day 7 as described in Fig. 1. Grey bars represent CD137 MFI in CD8^+^ tetramer positive T cells, red bars represent CD137 MFI in CD8^+^ tetramer negative T cells (bystander). ADO was omitted from donor #3 data due to low number of tetramer positive T cells. NLV, IVT, AVF and ATV represents NLVPMVATV, IVTDFSVIK, AVFDRKSDAK, and ATVQGQNLK, respectively. Data are representative of 4 (**A**) or 3 (**B**–**D**) independent experiments. Statistical analysis was performed as in Fig. 1 using median values of treated sample/peptide-only treated sample ratios (all comparisons within individual experiments). Log transformed median values were used in one-way ANOVA and subsequent Dunnett’s multiple comparison test relative to peptide-only treated samples. *adj p value ≤ 0.05; **adj p value ≤ 0.01; ***adj p value ≤ 0.001; ****adj p value ≤ 0.0001.

We next examined potential off-target effects on the expression of co-activating receptor CD137 on recently expanded memory T cells using this platform. We chose to focus on CD137 due to its ability to serve as a general marker for antigen-specific T cell activation regardless of cytokine secretion profile and T cell differentiation status^27^ (Fig. S12). The effect of drug on activation induced CD137 expression varied by recall antigen used (Fig. 2 and Fig. S13). 50 µM GS-5734 and 50 µM GS-441524 both increased memory T cell CD137 expression in donor #3 EBV-peptide stimulated PBMCs (Fig. 2B,C), whereas 50 µM GS-441524 treatment of donor #3 with AVF- and ATV-peptide stimulation resulted in significant CD137 reduction in bystander CD8^+^ T cells (Fig. 2D). This data shows that drug-induced activation (at least by this one measure) of memory T cells is peptide-dependent, and thus memory T cell subset-dependent. It also illustrates that in some cases, the activation status of antigen-specific memory T cells and bystander cells can further diverge due to drug.

## Discussion

We have described a simple drug screening platform that can be utilized for assessment of potential off-target memory T cell effects occurring from small molecule treatment. The recall antigen assay has been applied to test the efficacy of checkpoint blockade therapeutics as to whether they can further augment antigen-specific memory T cell recall^28–38^. However, to our knowledge, formatting the recall antigen assay to screen small molecule compounds is a novel application. We focused our efforts here on CD8^+^ memory T cells but this assay is amendable to studying CD4^+^ memory T cell off-target effects. In addition, this assay platform offers the advantage of utilizing unmanipulated donor PBMCs representing a heterogeneous mix of most immune cell types. It also offers the advantage of a simple physiological T cell stimulation and the opportunity to assay effects on bystander cells.

Use of persistent high dose adenosine provided a framework dosing regimen for screening compounds during the recall antigen assay. Adenosine administered only once 24-h after peptide stimulation (day 1) was ineffective in suppressing memory T cell expansion while adenosine administered at later time points (day 3 and day 5) was effective. This is in contrast with how early high dose adenosine treatment is able to inhibit proliferation of supraphysiologically stimulated T cells (^20^ and Fig. S1). Many factors could account for this difference including the homogeneity and differentiation state of T cells, the strength and diversity of receptors engaged during stimulation as well as proliferation kinetics. Our in vitro data shows that adenosine-mediated inhibition during recall is additive when adenosine is administered on both days 3 and 5. This persistent dosing regimen is needed to achieve maximum suppression and is likely due to the short half-life of adenosine^39^. Use of different compounds may necessitate different dosing regimens based on potency and half-life which.

Here we show the effects of high dose GS-5734 treatment on memory T cell expansion are consistent across different donors and peptide stimulations. GS-441524 showed the ability to negatively regulate antigen-specific T cell expansion in most donors screened. High dose GS-5734 consistently displayed cellular cytotoxicity whereas high dose GS-445124 had no observable effect on viability. In addition, both compounds elevated CD137 expression in EBV peptide stimulated memory T cells while effects on bystander cells was varied. Despite similarity between GS-5734 and GS-41524 in structure, their biological effects differed greatly. Given that GS-5734 is metabolized to intermediates including GS-445124^13^, the divergent effects between these two compounds is either tied to the direct action of GS-5734 and/or GS-5734 metabolites that are upstream of GS-445124. The results highlight the sensitivity of this assay to screen drugs of similar structures as to whether memory T cell proliferation and function is unencumbered. In theory, this assay is amenable to a variety of other drug classes such as those used in cancer immunotherapy^40^ to monitor for immune-related adverse events (irAEs). As a proof of concept, we utilized a structurally unrelated compound, JAK1/3 inhibitor Tofacitinib, and demonstrate complete suppression of antigen-specific memory T cell expansion without noticeable adverse effects on general cell viability (Fig. S11A,D). This result is consistent with the documented role Tofacitinib has in suppressing CD8^+^ T cell activity^41–43^.

We would predict enhanced utility of this assay by matching the recall peptide (disease model), “experienced” PBMCs and the appropriate investigational drug. For example, in the case of SARS-CoV-2 infection, one could stimulate SARS-CoV-2 “experienced” PBMCs with relevant peptide (e.g. from the spike protein) along with drug candidate to assess off-target effects specifically on spike-reactive memory T cells. In support of this, our data shows that the magnitude of GS-5734-mediated fold increase in memory T cells (mean of 1.4-fold for IVT and mean of 6.7-fold for ATV) and the magnitude of GS-441524-mediated fold decrease in memory T cells differs within the same donor using different viral peptide stimulations (Fig. 1D). This requires additional studies to understand if this phenomenon stems from intrinsic differences in antigens and the specific memory T cell subsets they activate and/or relates to existing antigen-specific memory T cell frequency as well as other factors.

## Supplementary Information

Supplementary Information 1.

Supplementary Information 2.
