# Supplementary material for: Adenosine-related small molecules show utility of recall antigen assay to screen compounds for off-target effects on memory T cells
Source: Sci Rep. 2021 May 5;11:9561. doi: 10.1038/s41598-021-88965-3 (PMC8100288; doi:10.1038/s41598-021-88965-3)
Supplement: Supplementary file 1 — Supplementary Information 1. [file 41598_2021_88965_MOESM1_ESM.docx]

**Adenosine-related Small Molecules Show Utility of Recall Antigen Assay to Screen Compounds For Off-Target Effects on Memory T cells**

Eden Kleiman, Gloria Sierra, Binchen Mao, Dennie Magcase, Marybeth V George, Pirouz M Daftarian

**SUPPLEMENTAL FIGURE LEGENDS**

**Supplementary Figure 1**. Adenosine reduces T cell proliferation. HLA-A*02:01 donor #1 PBMCs were first incubated with proliferation dye and then treated with anti-CD3/anti-CD28 beads with or without ADO on day 0 and 1 as described in Materials and Methods. Proliferation was assessed by flow cytometry on day 4. Three independent experiments are shown. Data were analyzed using Flowjo software proliferation modeling on live T cells. Right side of figure displays histogram plot window with division index, percent divided, expansion index and number of cells per division. Left side bar graph shows T cell division index with mean and SD. Ratio paired two-tailed t-test. **, p = 0.0025.

**Supplementary Figure 2**. Adenosine does not decrease Ki67^+^ MFI or the percentage of Ki67^+^ cells from the tetramer positive T cell fraction. HLA-A*02:01 PBMCs from donor #1 were treated with CMV pp65 NLVPMVATV ± ADO and assayed for tetramer positive cell Ki67 staining on Day 7 using the TrueNuclear Transcription Factor Staining kit. A) Flow plots depict representative data showing unstimulated (U), Peptide stimulated (P) and P + ADO. Bar graphs depict the B) percentage of tetramer positive T cells that are Ki-67 positive and C) Mean Fluorescence Intensity (MFI) of tetramer positive T cell Ki67 displayed as a ratio relative to peptide-treated only cells. Results are from 6 independent experiments. Unpaired two-tailed t-test. ***, p = 0.0007. One experiment from another HLA-A*02:01 donor (#2) yielded similar results (not shown).

**Supplementary Figure 3**. Cell viability is not significantly altered with drugs treatments, except for 50µM GS-5734. HLA-A*02:01 donor #1 PBMCs treated with CMV pp65 NLVPMVATV along with various other treatments were assessed on day 7 by flow cytometry. A) FACS plots illustrating the scatter of cells from day 7 cultured cells. 50µM GS-5734 (Rem) results in significant depletion of the lymphocyte gate and corresponding accumulation of cellular debris on the bottom left side of axis. GS-441524 (Parent) does not result in increased cell death. B) The percentage of cellular debris (among total cells) was assessed from Day 7 cultured cells. Data is from 4 independent experiments with 2-3 replicates for each experiment. One-way ANOVA and subsequent Dunnett’s multiple comparison test was performed on debris or lymphocyte % values relative to peptide-only treated samples. ****, adj p value ≤ 0.0001.

**Suppelmentary Figure 4**. 50µM GS-5734 causes significant increase in cell death. HLA-A*11:01 donor #3 PBMCs treated with 10µg/mL of either A) EBV EBNA-3B peptide IVTDFSVIK, B) EBV EBNA4 peptide AVFDRKSDAK, or C) CMV pp65 peptide ATVQGQNLK along with various other treatments as in Supplementary Figure 3. The percentage of cellular debris (among total cells) was assessed from Day 7 cultured cells. Data is from 3 independent experiments with 2-3 replicates for each experiment. One-way ANOVA and subsequent Dunnett’s multiple comparison test relative to peptide-only treated samples as in Supplementary Figure 3. *, adj p value ≤ 0.05; **, adj p value ≤ 0.01.

**Supplementary Figure 5.** Cellular toxicity of drug treatments. HLA-A*02:01 donor #1 PBMCs treated overnight with indicated drugs or DMSO vehicle. No peptide stimulation was used for this experiment. Indicated cell fraction (y axis label) was analyzed by Annexin V (AnV) vs 7AAD co-staining. Displayed are the single positive AnV cells (grey bars – early apoptosis) as well as the AnV positive, 7AAD double positive cells (black bars – late stage apoptosis). Data is from 3 independent experiments with 2 replicates for each experiment. Ratios of all combinations of treated sample relative to unstimulated sample were calculated within each experiment. Median ratio values for each experiment are plotted in graphs. Log transformed median values were analyzed by one-way ANOVA and subsequent Dunnett’s multiple comparison test relative to unstimulated. *, adj p value ≤ 0.05; **, adj p value ≤ 0.005; ***, adj p value ≤ 0.0005; ****, adj p value ≤0.0001.

**Supplementary Figure 6**. ADO inhibits antigen-specific memory T cell recall and can be rescued by adenosine deaminase enzyme supplementation, and ADO breakdown metabolite inosine does not inhibit recall. A) HLA-A*02:01 donor #1 PBMCs treated either with peptide alone (CMV pp65 NLVPMVATV), peptide + ADO, peptide + 10µg/mL adenosine deaminase (ADA) or a combination of peptide + ADO + ADA. Results from 4 separate experiments with 2 replicates per experiment B) Recall antigen assay experimental setup as in A, comparing addition of ADO versus ADO metabolite inosine (INO), both at the same concentrations (1mM final on day 0, 0.5mM final on days 3 and 5). Results are from 2 independent experiments and are expressed as fold change (FC) relative to peptide-alone stimulated samples. Within each experiment, all combination ratios of treated sample relative to peptide-only treated sample were calculated and the median ratio value for each experiment is plotted. Log transformed median values were analyzed by one-way ANOVA and subsequent Dunnett’s multiple comparison test relative to peptide-treated only samples. **, adj p value = 0.0059; ****, adj p value ≤ 0.0001.

**Supplementary Figure 7**. Adenosine-mediated alterations in co-inhibitory receptor expression. HLA-A*02:01 donor #1 PBMCs treated with 2.5µg/mL CEF peptide pool (ProMix CEF Peptide Pool, Proimmune Ldt) containing CMV pp65 NLVPMVATV for 2 experiments or 10µg/mL pure CMV pp65 NLVPMVATV peptide for 2 experiments. Cells were treated with either peptide/peptide cocktail (P) alone or with ADO. In these early experiments, ADO was administered on days 0, 2, 4 and 6 at 1mM final day 0 and 0.5mM final on days 2, 4 and 6. Surface expression of co-inhibitory receptors A) PD-1, B) LAG-3 and C) TIM-3 were assessed on CMV pp65 NLVPMVATV-specific CD8^+^ T cells or tetramer negative CD8^+^ T cells on day 7. Results are from 4 independent experiments. Two-tailed paired t-test analysis. *, p ≤ 0.05.

**Supplementary Figure 8**. Adenosine stimulation increased the percentage of CD14^+^ CD11b^-^ cells and their surface expression of PD-1 co-inhibitory receptor ligands PD-L1/PD-L2 in donor #1. HLA-A*02:01 donor #1 PBMCs treated with or without 1mM ADO once for either 24 hours or 48 hours. Cells were assayed for (A-B) the percentage of CD14^+^, CD11b^-^ cells and (C-D) the MFI surface expression of PD-L1/PD-L2 (both proteins on the same PE channel) on CD14^+^, CD11b^-^ cells. For C-D, grey bars depict the PD-L1/PD-L2 expression levels on CD14^+^ CD11b^-^ cells while black bars depict PD-L1/PD-L2 expression on T cells for comparison. Results are from 2 independent experiments for each time point. Two-tailed unpaired t-test analysis of CD14^+^ CD11b^-^ cell data. **, p ≤ 0.01; ***, p ≤ 0.001; ****, p ≤ 0.0001. Results from CD14^+^ CD11b^+^ cells did not show a significant difference in MFI (data not shown).

**Supplementary Figure 9**. Adenosine stimulation does not reduce IFNγ production or activation induced TOX expression on previously expanded memory T cells re-stimulated with peptide. HLA-A*02:01 donor #1 PBMCs that were previously expanded (with NLVPMVATV peptide at least 7 days) were combined with unexpanded PBMCs of the same donor at a ratio of 2:1 on day 0. Cell co-cultures were either not re-stimulated or re-stimulated with 10µg/mL NLVPMVATV peptide (P) for 48 hours. Additionally, 1mM ADO was either added or not as indicated at the same time as peptide re-stimulation. A) representative plots showing IFNγ and TOX expression among tetramer positive T cells, except for black dots which are from CD3- cells. IFNγ plots with y axis tetramer and CD3 are meant to illustrate activation due to internalization of TCR (tetramer staining) and CD3. B-E) Bar graphs depicts IFNγ and TOX MFI and percentage of positivity within the tetramer^+^ T cell gate either stimulated with peptide (grey bars) or non-peptide-stimulated (black bars). Because tetramer positive T cells from donor #1 are only detectable by flow after day 5 post-peptide stimulation, this data is representative of the IFNγ and TOX expression that was induced 48 hours post-peptide stimulation on tetramer positive cells already present (expanded) as opposed to unexpanded PBMCs which will not have detectable tetramer positive cells at this time point. Staining was performed using TrueNuclear Transcription Factor kit with Brefeldin A treatment 4 hours prior to staining. Results are from 3 independent experiments. Data depicted as bar graphs but two-tailed paired t-test analysis was performed using paired data points from each individual experiment. Statistical analysis only shown for peptide treated samples (grey bars), black bars displayed for reference. *, p = 0.01.

**Supplementary Figure 10**. Antigen-specific T cell recall is altered at high doses of GS-5734 and parent drug GS-441524. HLA-A*02:01 donor #1 PBMCs treated with CMV pp65 NLVPMVATV and assayed for tetramer positive cells on Day 7 as described in Figure 1C. ADO dissolved in complete media is denoted as ADO^aq^. ADO dissolved in DMSO (ADO^DMSO^), GS-5734 (DMSO) and parent drug GS-445124 (DMSO) were all given at ascending doses of 500nM, 5µM or 50µM. DMSO vehicle control was administered at 0.1%, 0.01% or 0.001% final. GS-5734 at 50µM is depicted as a grey striped bar to denote the toxicity observed. NECA and CGS-21680 (DMSO) were both given at ascending doses of 100nM, 1µM or 10µM. All treatment sample data are normalized to peptide-only samples within each experiment (comparison using horizontal line at 1 on y axis). Data is from 4 independent experiments with 2 replicates per condition. Ratios of all combinations of treated samples relative to peptide-only treated sample were calculated within individual experiments and the median ratio value for each experiment is plotted. Log transformed median values were used in one-way ANOVA and subsequent Dunnett’s multiple comparison test relative to peptide-treated only samples. **, adj p value = 0.0013; ****; adj p value ≤ 0.0001.

**Supplementary Figure 11**. Two additional donors show consistent high dose GS-5734 effects on antigen-mediated memory T cell expansion while high dosage of parent GS-445124 drug shows donor to donor variability effect on memory T cell expansion. A,D) Donor #4 and donor #5, both HLA-A*02:01, were stimulated as described in Figure 1C. Additionally, 0.1µM or 1 µM Tofacitinib was given at the same dosing schedule. High dose GS-5734 is depicted as a grey striped bar as previously described. Left Y axis is the percent of CD3^+^ T cells that are tetramer positive. Right Y axis is for the percentage of singlet cells that are viable (green dots). These samples were co-stained for surface CD28 expression (B, C, E, F). Both CD28 MFI (B, E) and CD28 percent positive (C, F) are shown for tetramer positive T cells, CD8^+^ tetramer negative T cells and non-T cells (CD3^-^). Results are from 1 independent experiment for each donor performed in triplicate.

**Supplementary Figure 12**. CD137 co-stimulatory surface marker used as a surrogate for activation status on day 7 of recall. Displayed is data from HLA-A*02:01 donor #1 PBMCs treated with peptide only (NLVPMVATV) as a representative. CD137 expression non-overlaid histograms (bottom left) for CD3 negative cells (orange), CD3^+^ CD8^+^ tetramer negative T cells (red) or CD3^+^ CD8^+^ tetramer positive T cells (blue). Overlay plots include CD3, tetramer, 7AAD viability dye, CD4, CD8 and human memory T cell marker CD45RO.

**Supplementary Figure 13**. Effect of drug on T cell activation. HLA-A*02:01 donor #1 PBMCs were treated as in Figure 1C and assayed for CD137 on day 7 as described in Figure 2. Grey bars represent CD137 MFI in tetramer positive T cells. Data are representative of 4 independent experiments with 2 replicates for each condition. For statistical analysis, ratio of all combinations of treated samples relative to peptide-only treated samples within each experiment were calculated and the median ratio value within each experiment was log transformed and used in one-way ANOVA and subsequent Dunnett’s multiple comparison test relative to peptide-only treatment.
