# Supplementary material for: Adenosine-related small molecules show utility of recall antigen assay to screen compounds for off-target effects on memory T cells
Source: Sci Rep. 2021 May 5;11:9561. doi: 10.1038/s41598-021-88965-3 (PMC8100288; doi:10.1038/s41598-021-88965-3)

Adenosine-related Small Molecules Show Utility of Recall Antigen Assay to Screen Compounds For Off-Target Effects on Memory T cells

Eden Kleiman, Gloria Sierra, Binchen Mao, Dennie Magcase, Marybeth V George, Pirouz M Daftarian

Supplementary Figure 1

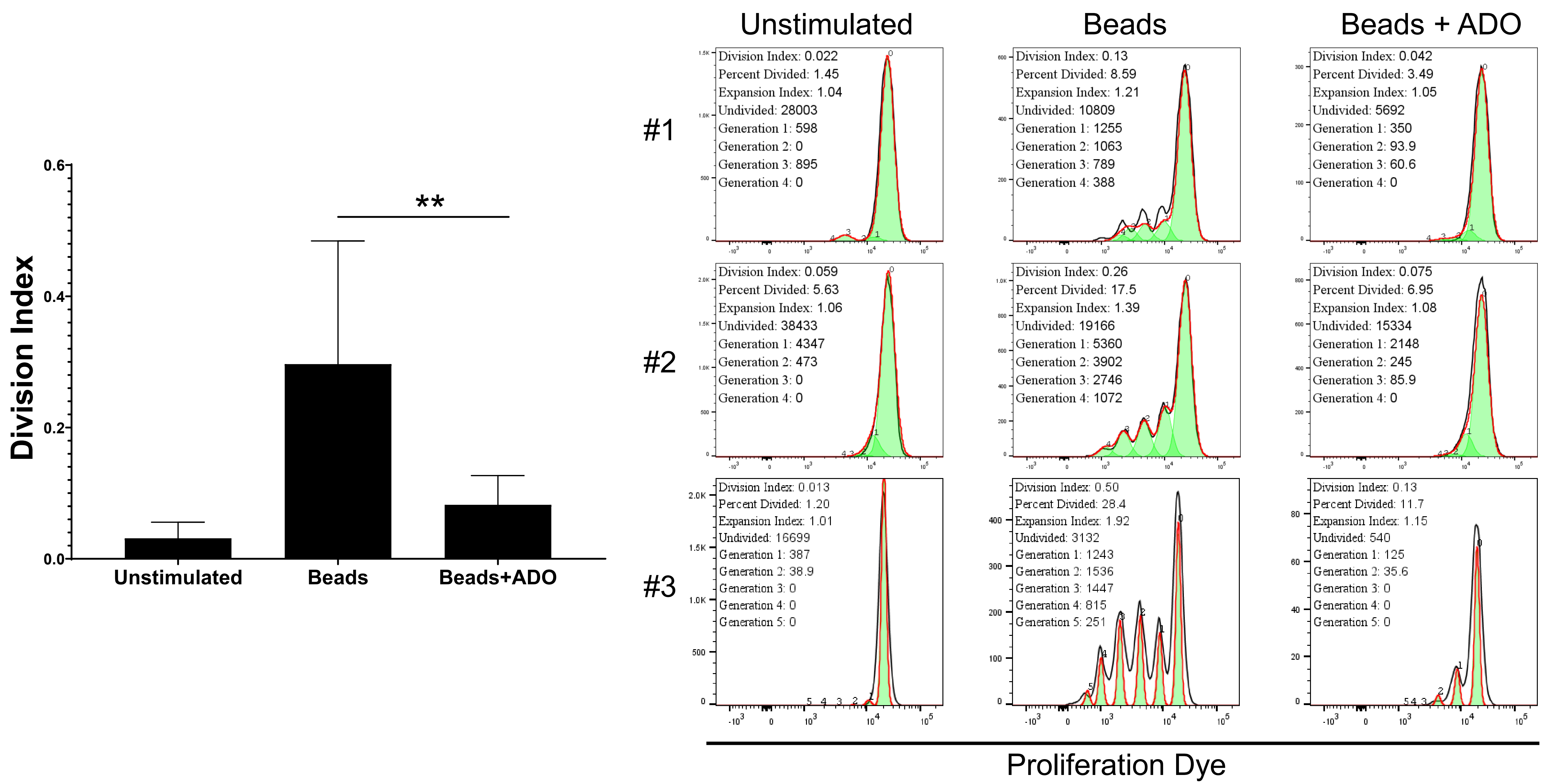

Supplementary Figure 2

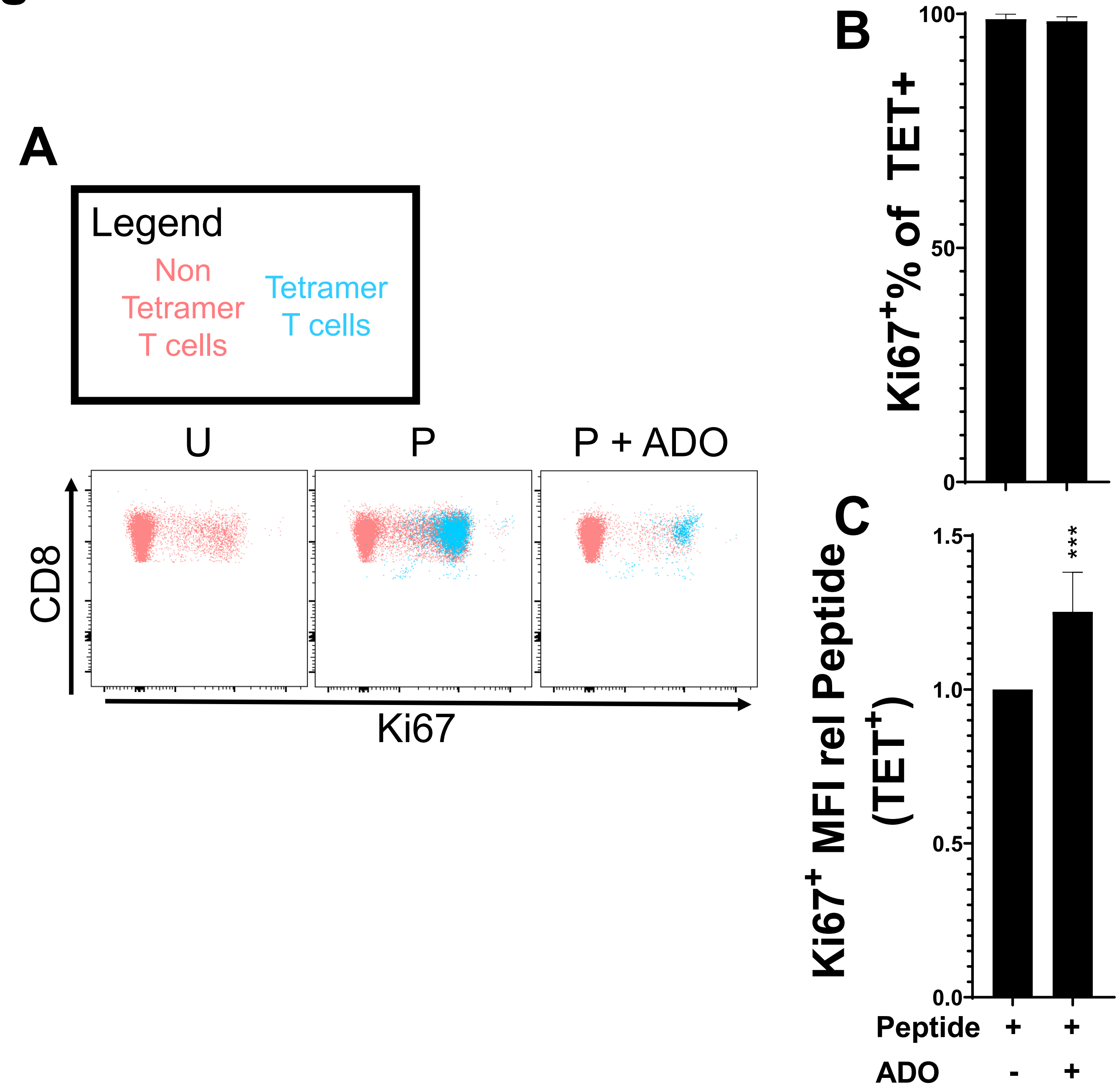

Supplementary Figure 3

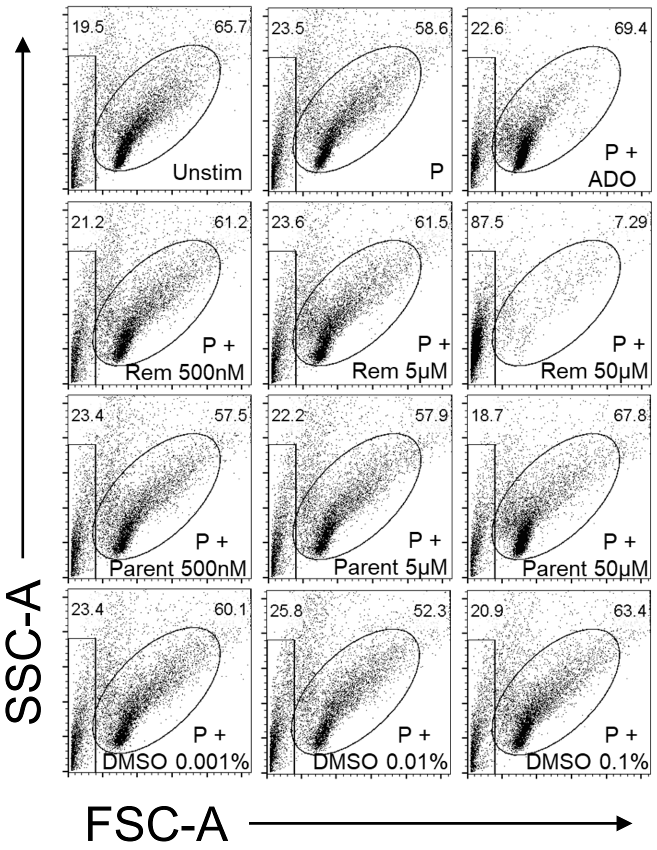

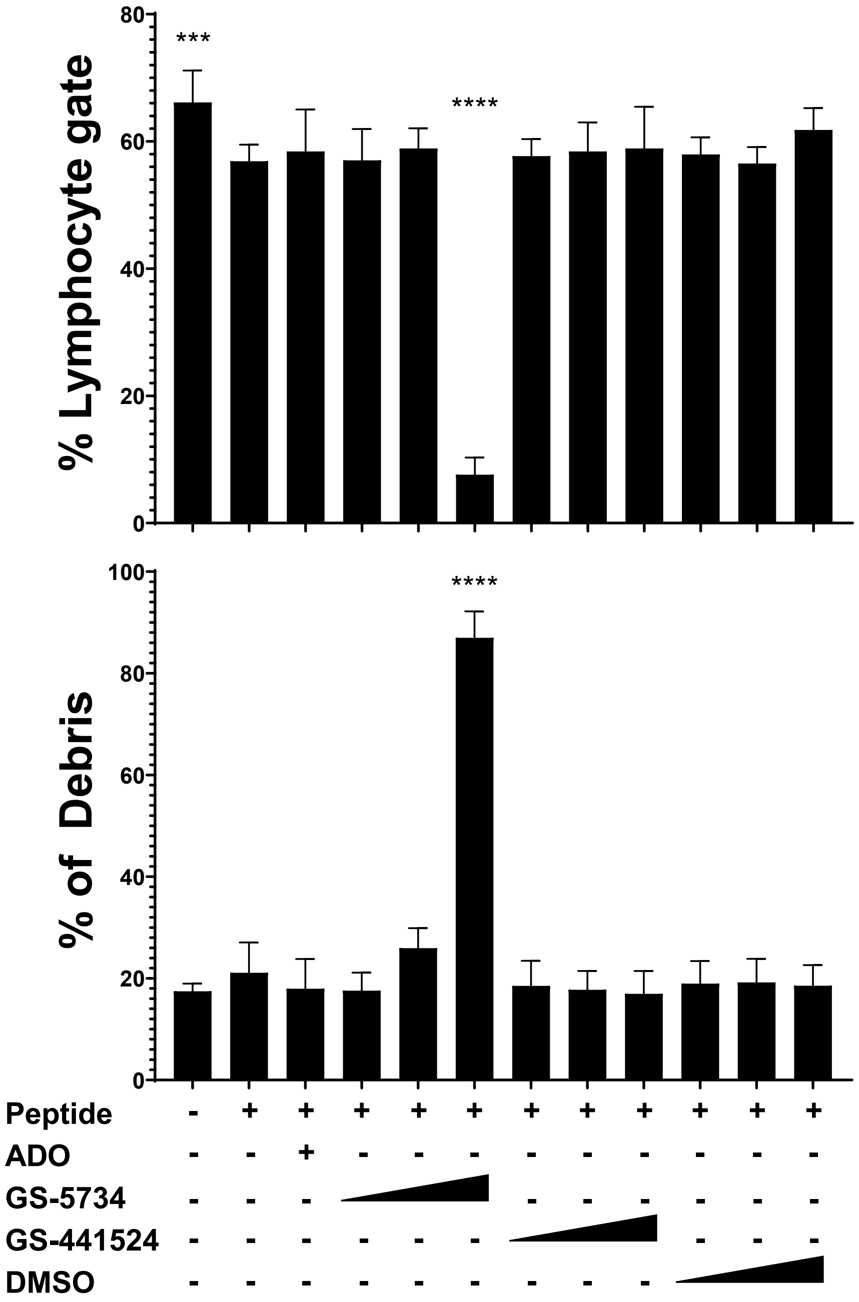

Supplementary Figure 4

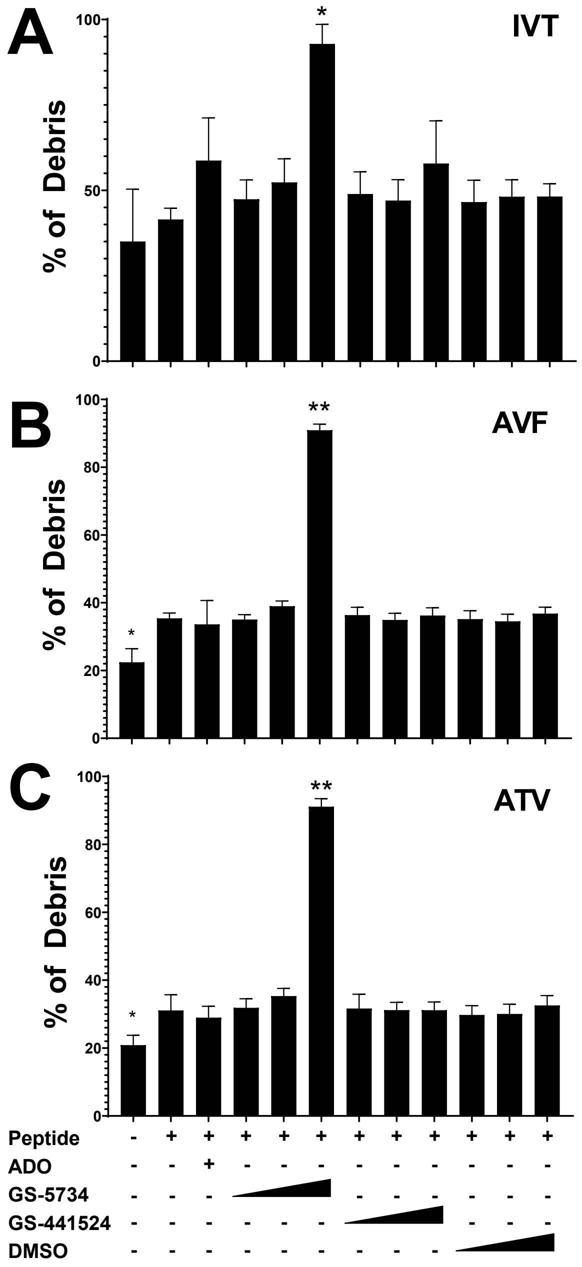

Supplementary Figure 5

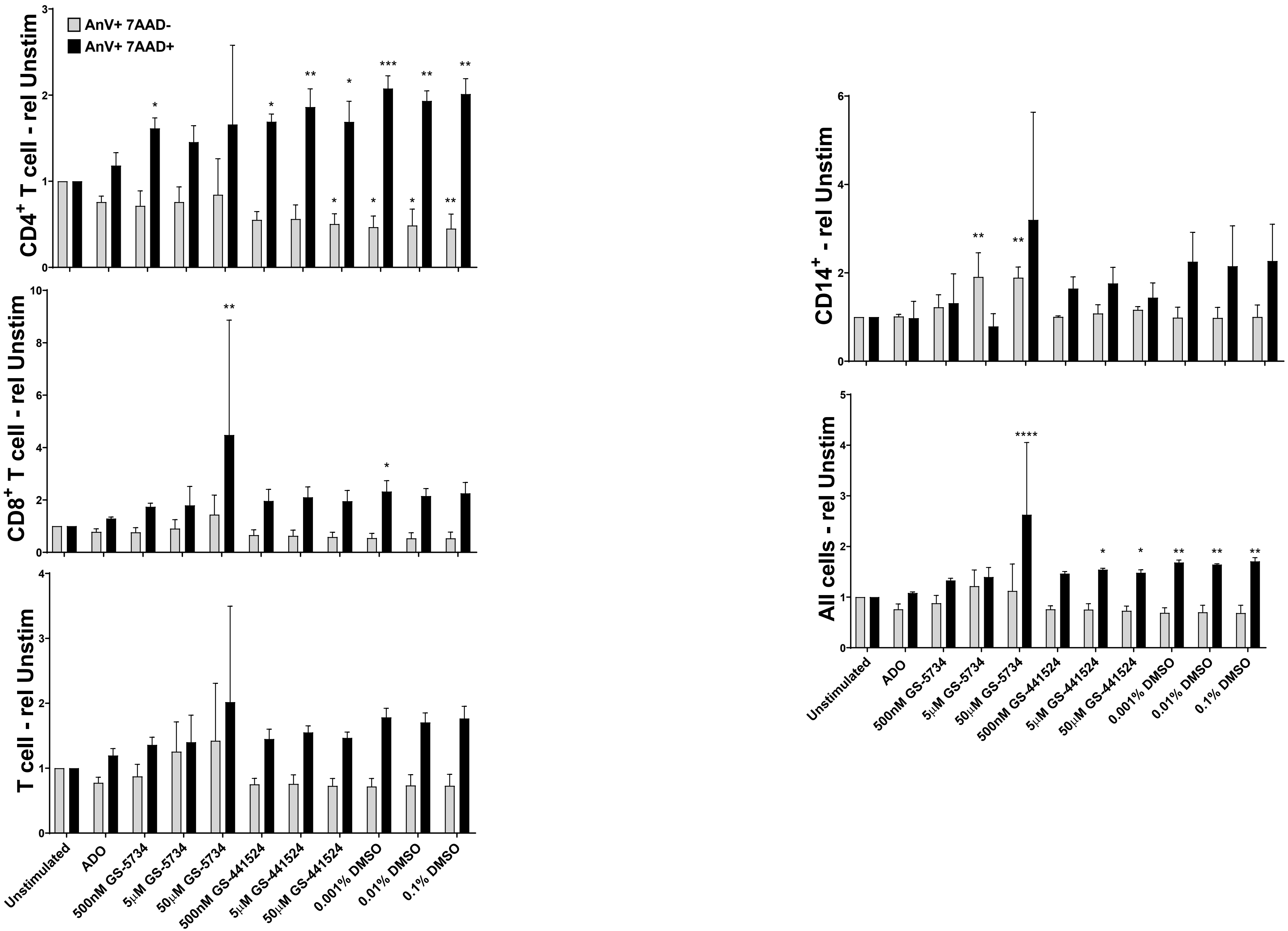

Supplementary Figure 6

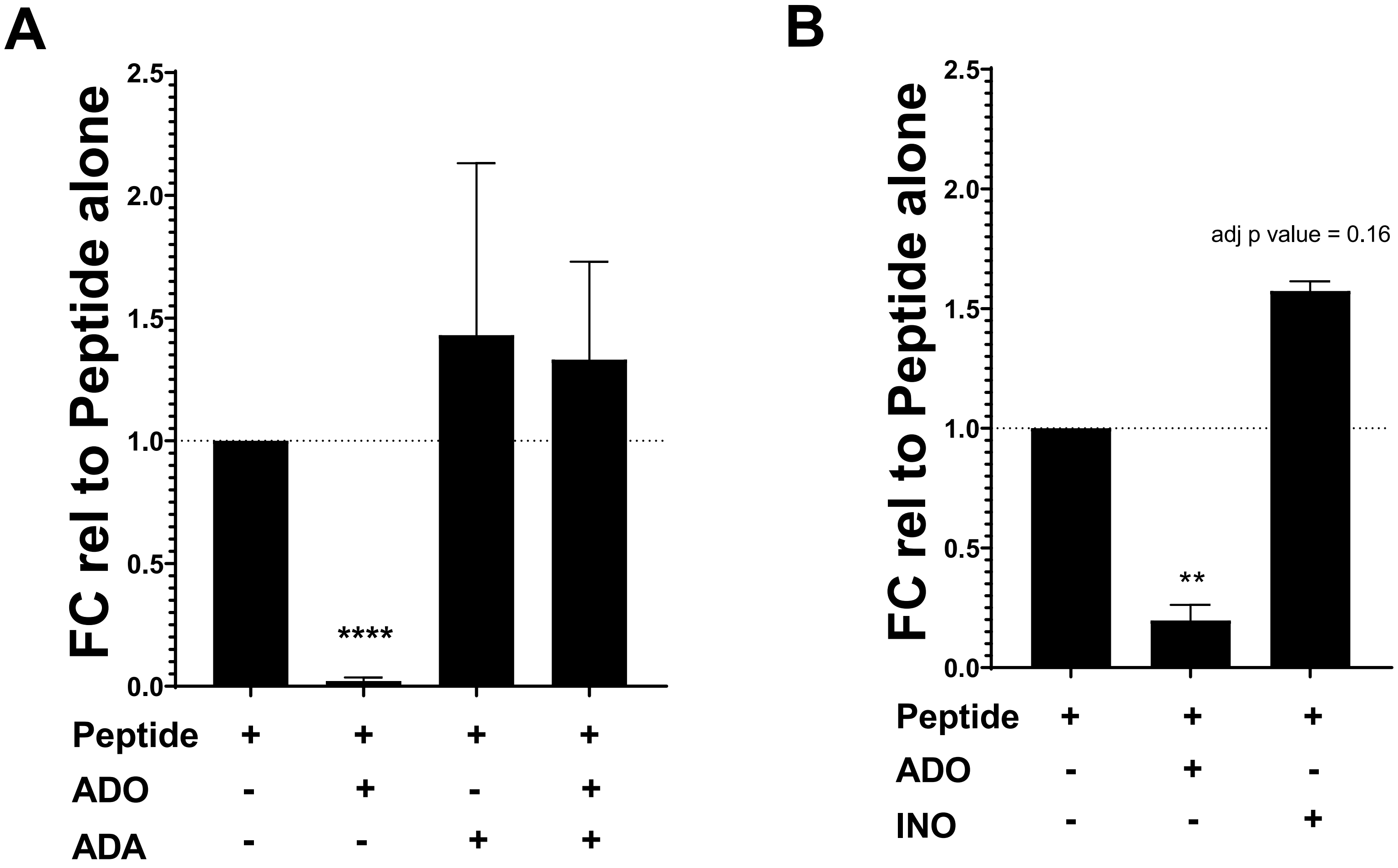

Supplementary Figure 7

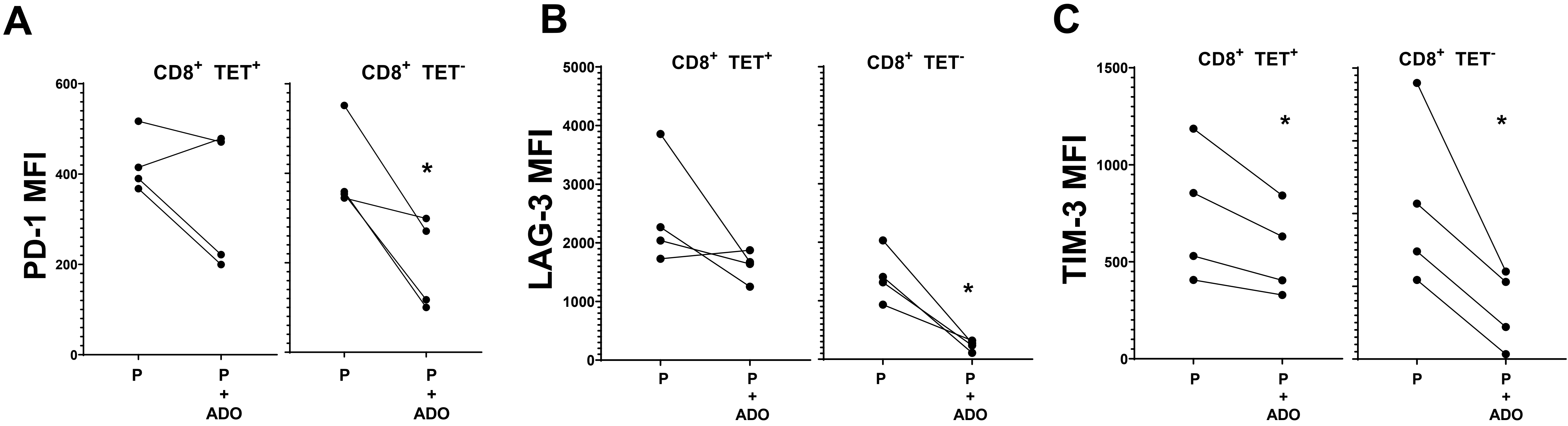

Supplementary Figure 8

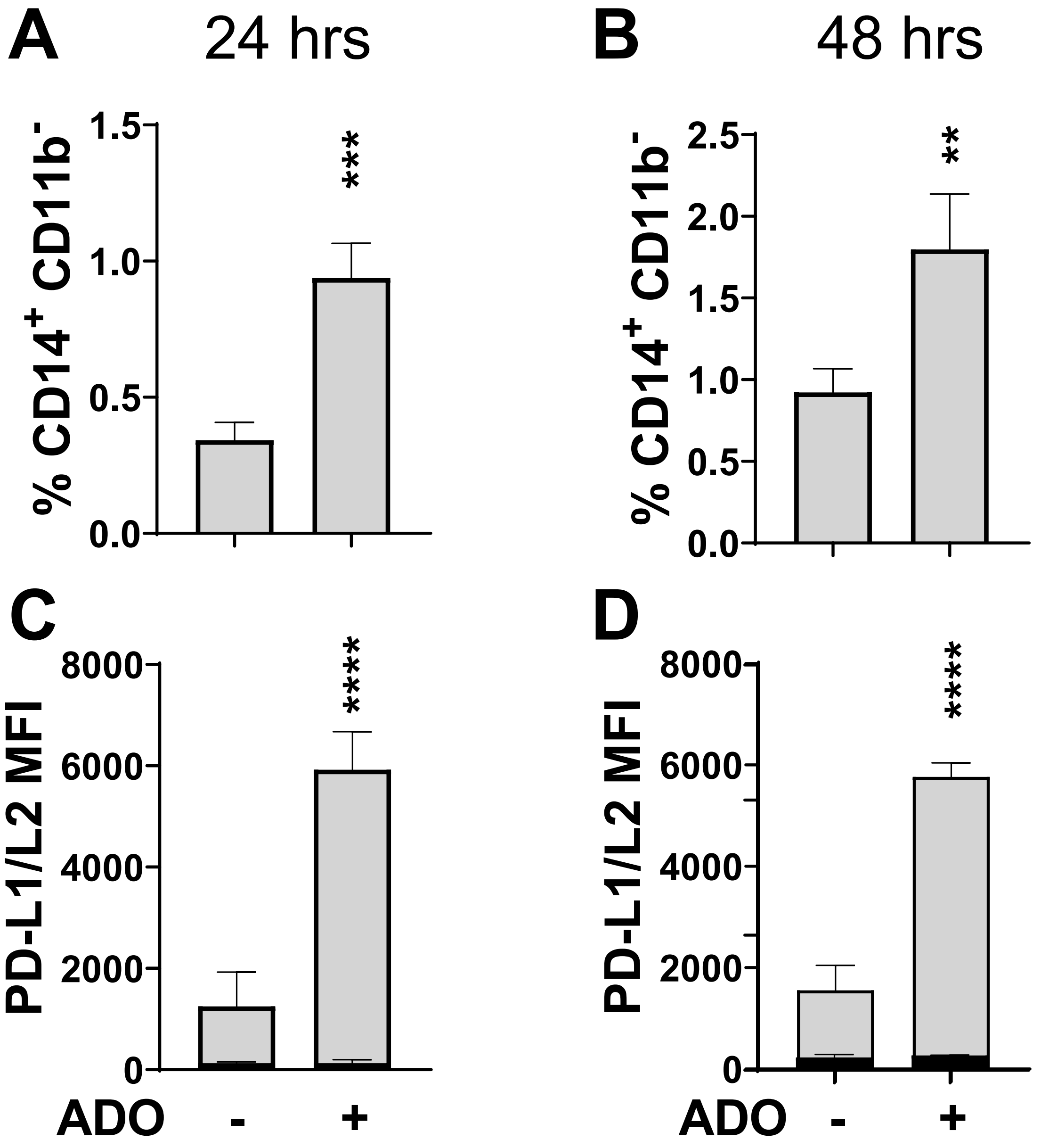

Supplementary Figure 9

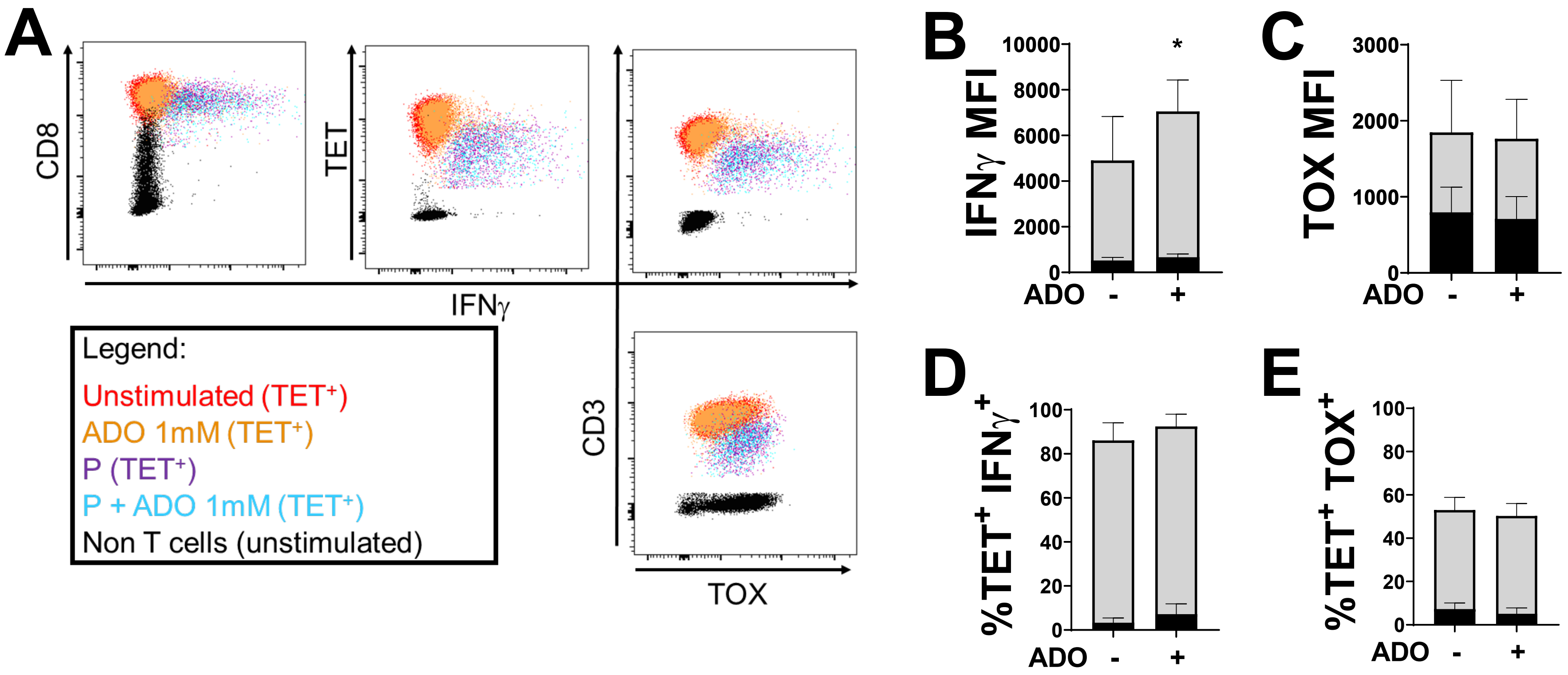

Supplementary Figure 10

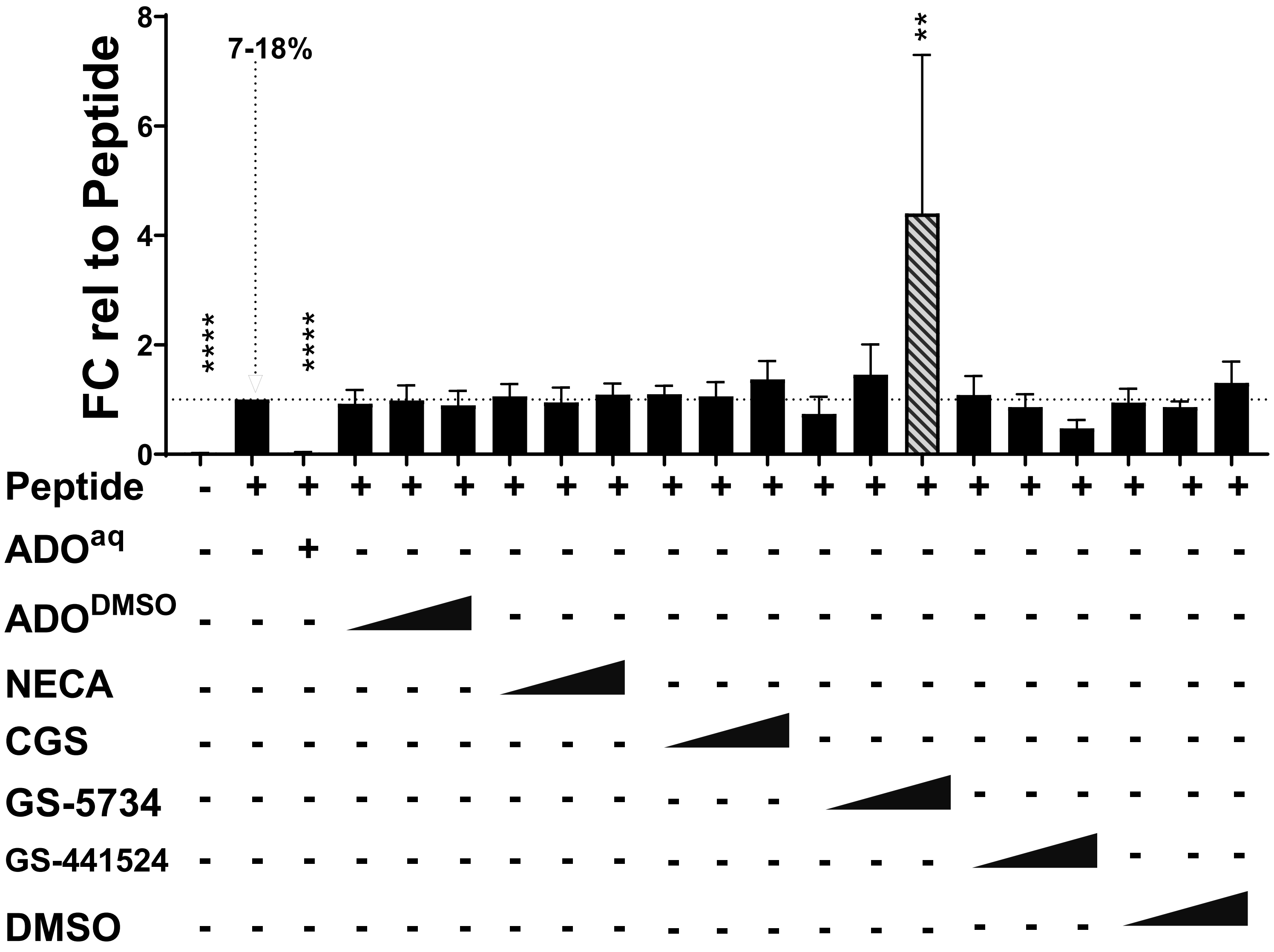

Supplementary Figure 11

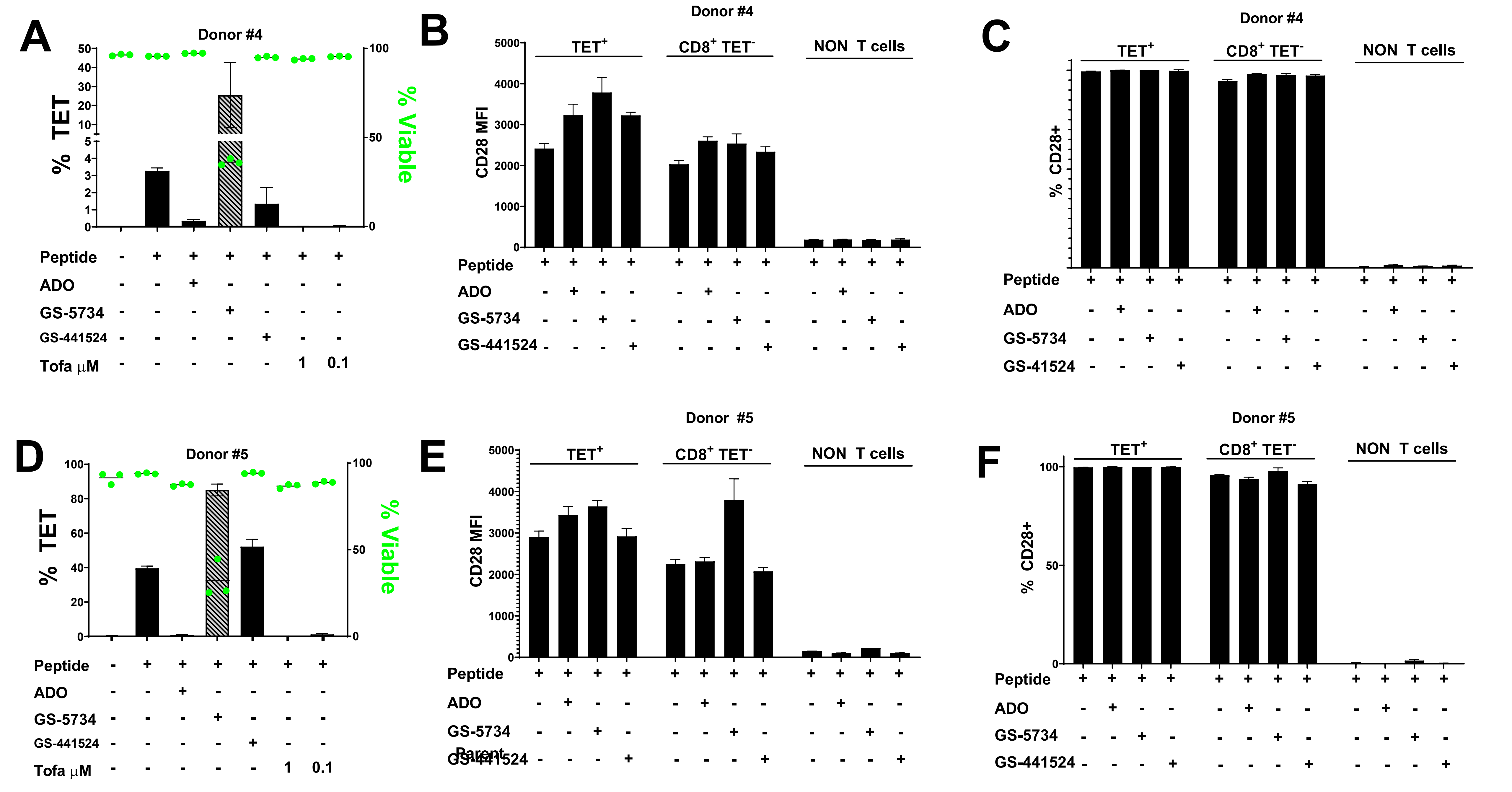

Supplementary Figure 12

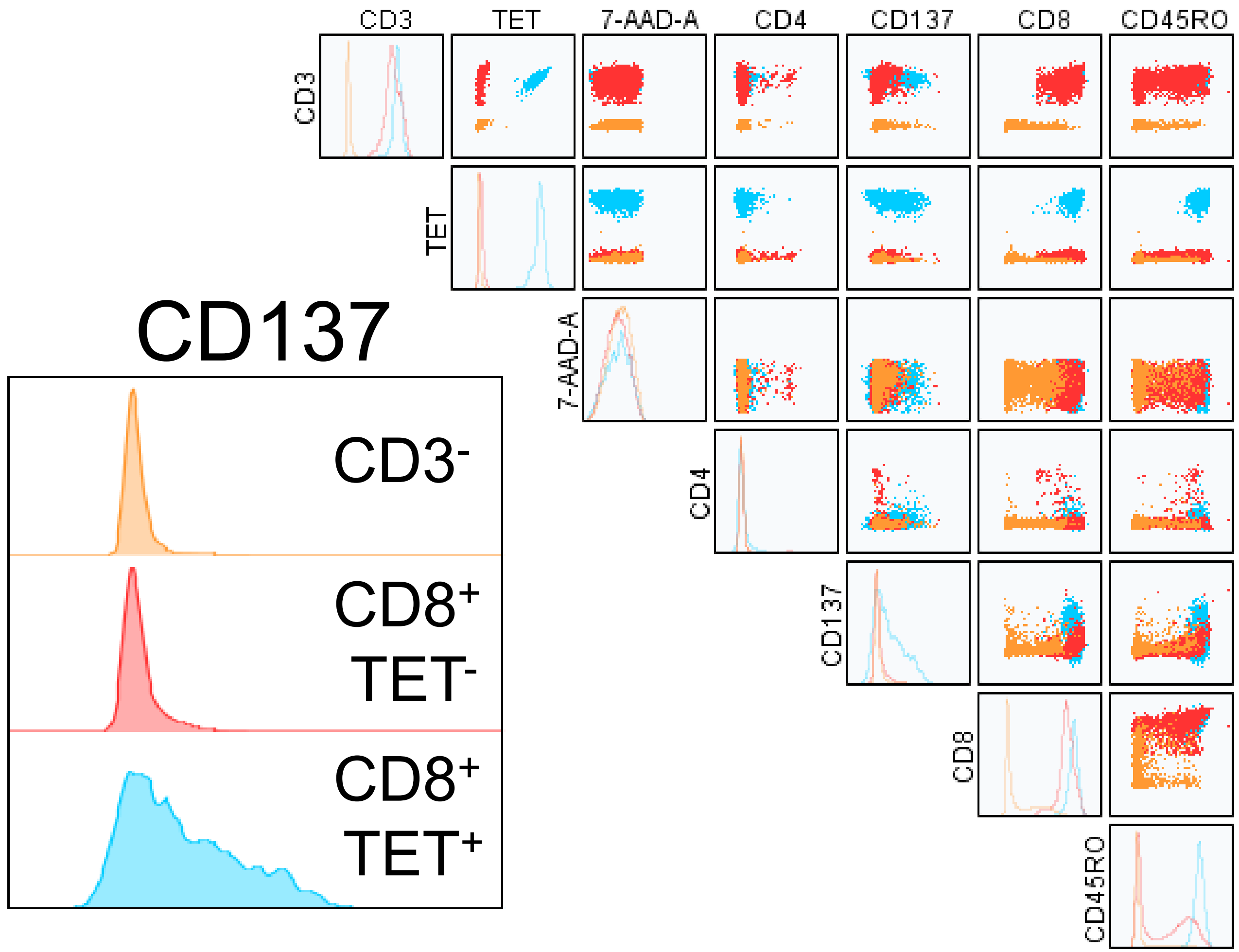

Supplementary Figure 13

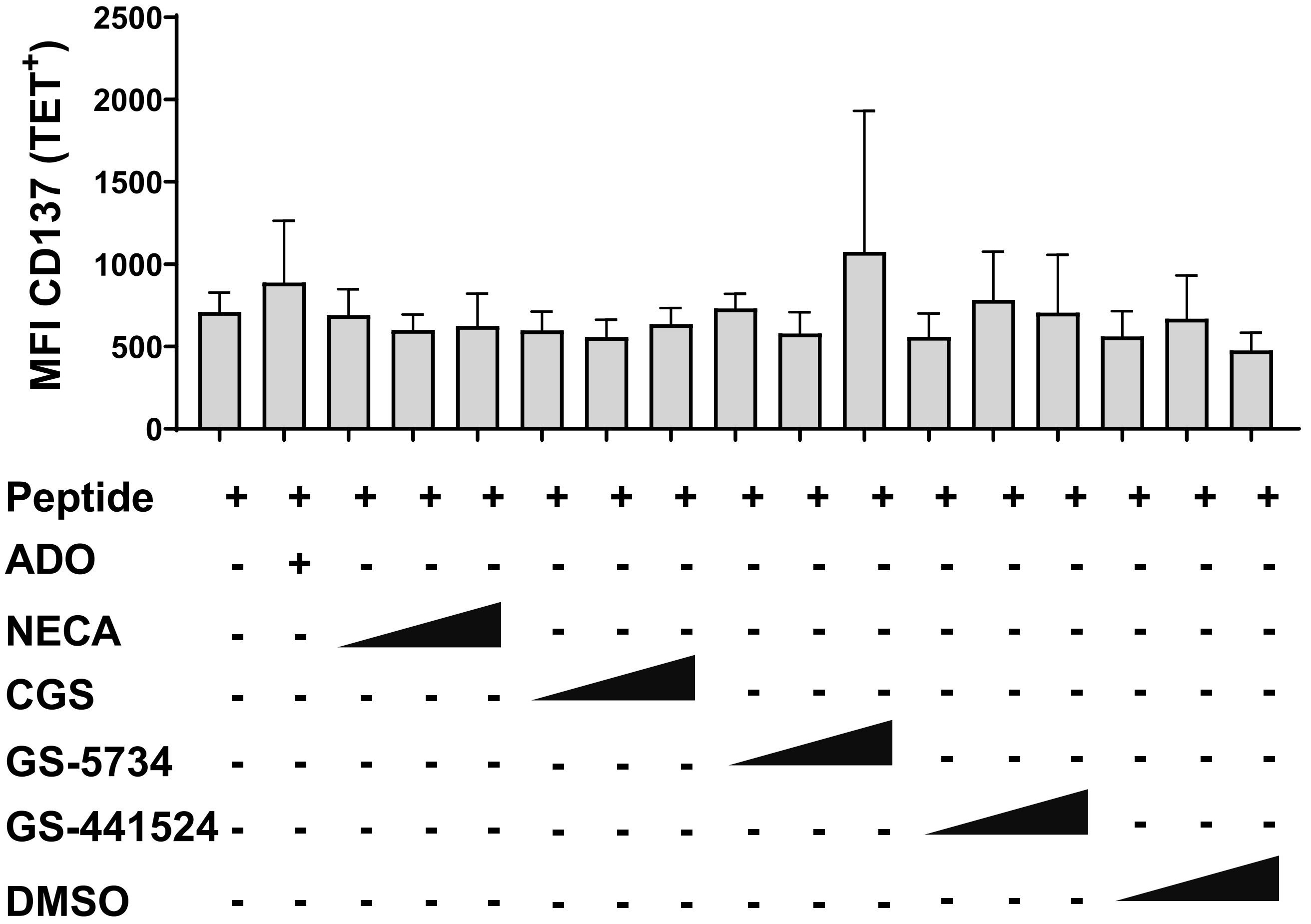

Supplement: Supplementary file 2 — Supplementary Information 2. [file 41598_2021_88965_MOESM2_ESM.pdf]
